# A Phase II Study Evaluating the Interest to Combine UCPVax, a Telomerase CD4 T_H_1-Inducer Cancer Vaccine, and Atezolizumab for the Treatment of HPV Positive Cancers: VolATIL Study

**DOI:** 10.3389/fonc.2022.957580

**Published:** 2022-07-19

**Authors:** Magali Rebucci-Peixoto, Angélique Vienot, Olivier Adotevi, Marion Jacquin, Francois Ghiringhelli, Christelle de la Fouchardière, Benoit You, Tristan Maurina, Elsa Kalbacher, Fernando Bazan, Guillaume Meynard, Anne-Laure Clairet, Christine Fagnoni-Legat, Laurie Spehner, Adeline Bouard, Dewi Vernerey, Aurélia Meurisse, Stefano Kim, Christophe Borg, Laura Mansi

**Affiliations:** ^1^ Department of Oncology, Centre Hospitalier Universitaire, Besançon, France; ^2^ Clinical Investigational Center, CIC-1431, Centre Hospitalier Universitaire, Besançon, France; ^3^ INSERM, EFS BFC, UMR1098 RIGHT, University of Bourgogne Franche-Comté, Besançon, France; ^4^ Cancéropôle Est, Strasbourg, France; ^5^ Department of Oncology, Centre Georges-François Leclerc, Dijon, France; ^6^ Department of Oncology, Centre Léon Bérard, Lyon, France; ^7^ Department of Oncology, Hospices Civils de Lyon, Lyon, France; ^8^ Department of Pharmacy, University Hospital of Besançon, Besançon, France; ^9^ Methodology and Quality of Life in Oncology Unit, Centre Hospitalier Universitaire de Besançon, Besançon, France; ^10^ Department of Oncology, Sanatorio Allende, Cordoba, Argentina

**Keywords:** HPV-related cancer, anal carcinoma, head and neck, cervix, atezolizumab, immunotherapy, vaccine

## Abstract

**Background:**

There is a strong rational of using anti–programmed cell death protein-1 and its ligand (anti–PD-1/L1) antibodies in human papillomavirus (HPV)–induced cancers. However, anti–PD-1/L1 as monotherapy induces a limited number of objective responses. The development of novel combinations in order to improve the clinical efficacy of an anti–PD-1/L1 is therefore of interest. Combining anti–PD-1/L1 therapy with an antitumor vaccine seems promising in HPV-positive (+) cancers. UCPVax is a therapeutic cancer vaccine composed of two separate peptides derived from telomerase (hTERT, human telomerase reverse transcriptase). UCPVax is being evaluated in a multicenter phase I/II study in NSCLC (non–small cell lung cancer) and has demonstrated to be safe and immunogenic. The aim of the VolATIL study is to evaluate the combination of atezolizumab (an anti-PD-L1) and UCPVax vaccine in a multicenter phase II study in patients with HPV^+^ cancers.

**Methods:**

Patients with HPV^+^ cancer (anal canal, head and neck, and cervical or vulvar), at locally advanced or metastatic stage, and refractory to at least one line of systemic chemotherapy are eligible. The primary end point is the objective response rate (ORR) at 4 months. Patients will receive atezolizumab every 3 weeks at a fixed dose of 1,200 mg in combination with the UCPVax vaccine at 1 mg subcutaneously.

**Discussion:**

Anti-cancer vaccines can restore cancer-immunity *via* the expansion and activation of tumor-specific T cells in patients lacking pre-existing anti-tumor responses. Moreover, preclinical data showed that specific T_H_1 CD4 T cells sustain the quality and homing of an antigen-specific CD8^+^ T-cell immunity. In previous clinical studies, the induction of anti-hTERT immunity was significantly correlated to survival in patients with advanced squamous anal cell carcinoma. Thus, there is a strong rational to combine an anti-cancer hTERT vaccine and an immune checkpoint inhibitor to activate and promote antitumor T-cell immunity. This pivotal proof of concept study will evaluate the efficacy and safety of the combination of a telomerase-based T_H_1 inducing vaccine (UCPVax) and an anti–PD-L1 (atezolizumab) immunotherapy in HPV^+^ cancers, as well as confirming their synergic mechanism, and settling the basis for a new combination for future clinical trials.

**Clinical Trial Registration:**

https://www.clinicaltrials.gov/, identifier NCT03946358.

## 1 Introduction

More than 90% of anal and cervical cancers and about 70% of oropharyngeal cancers are linked to the human papillomavirus (HPV) infection (1, 2), and their incidence is steadily increasing (3, 4). HPV oncogenic proteins are trans-activators of telomerase (5). Telomerase is an enzyme that is re-expressed in most tumor cells and it ensures the reconstitution of the telomeres, which are shortened at each cell division and prevents the entry of the cell in replicative senescence. This enzyme is composed of a catalytic subunit, human telomerase reverse transcriptase (hTERT) and a structural subunit composed of a telomerase RNA component (TERC). The main mechanism of telomere maintenance in cancers depends on the reactivation of hTERT, which is overexpressed in more than 90% of human cancers, thus conferring to cancer cells a form of immortality (6).

This overexpression of telomerase in tumor cells compared with normal cells confers to hTERT the property of a tumor antigen. Our group has conducted a clinical trial (Epitopes-HPV01 and Epitopes-HPV02) in advanced squamous cell anal cancer where a correlation was evidenced between the presence of anti-HPV immunity and anti-telomerase T_H_1 CD4 T-cell responses. We could unwaveringly establish that telomerase is an appropriate antigen in HPV-related cancers (7, 8).

Besides, tumor-reactive CD4^+^ T cells have been found to ensure efficient effector cytolytic T lymphocytes (CTLs) recruitment at the tumor site (9, 10). In consequence, the promotion of tumor specific T_H_1 CD4 activation might be an attractive therapeutic option to enhance anti–programmed cell death protein-1 and its ligand (anti–PD-1/L1) efficacy (11). However, no option is currently available to expand tumor specific T_H_1 lymphocytes in most patients. We have used an optimized reverse immunology approach to identify four novel MHC class II–restricted peptides derived from hTERT referred as “Universal Cancer Peptides” (UCP). UCPVax is a therapeutic cancer vaccine composed of two separate peptides called UCP2 and UCP4 derived from telomerase (12). This UCPVax vaccine is currently being evaluated in a multicenter phase I/II study in NSCLC (NCT2818426). Results from the phase I study indicated that UCPVax was safe, well tolerated, and immunogenic at the doses and schedule tested, and 1 mg of each peptide was recommended for phase II. UCPVax was immunogenic in 60% of NSCLC patients at least after three injections, with preliminary signals of clinical efficacy in this population. Accordingly, we selected this 1-mg dose of each peptide in the VolATIL study.

Preclinical and clinical data demonstrated PD-1/L1 immune checkpoint as a relevant target for immunotherapy in HPV^+^ cancers, based on the prominent role of PD-1 and PD-L1 in HPV-driven immune evasion. The role of PD-L1 in immune evasion of HPV-associated tumors was well described in head and neck squamous cell carcinoma (HNSCC). A membranous expression of PD-L1 on epithelial cells and macrophages was observed in tonsillar crypts of HPV-infected patients. Similarly, the majority of CD8^+^ T lymphocytes of these patients expressed high levels of PD-L1 (13–15). In mice, PD-1 neutralization synergized with HPV vaccine to promote an anti-tumor immunity (16).

Recent clinical trials also confirmed the interest of PD-1/L1 inhibitors in HPV^+^ cancers. In patients with advanced squamous cell carcinoma of the anus (SCCA), refractory to at least one line of treatment, five prospective phase I/II trials included 298 patients (11, 17–21). ORR was 13.4% including the 3.7% of complete responses. The median overall survival (OS) was approximately 11 months (**Table 1**). In cervical cancer patients with PD-L1–positive advanced disease and refractory to at least one systemic treatment, pembrolizumab induced an ORR in 15 of 101 (14.9%) patients including two (2%) complete responses in two prospective trials (22, 23). In HNSCC, the strongest available data for checkpoint inhibitors in refractory disease come from an initial and expansion cohort of a phase Ib study using pembrolizumab in the recurrent/metastatic setting. Among 192 patients enrolled, 18% presented an objective response including four complete responses (24).

**Table 1:** PD1/L-1 treatment in patients with refractory-advanced squamous cell carcinoma of the anus.

Treatment(Reference)	Nivolumab (15)	Pembrolizumab (16, 17)	Retifanlimab (18)	Avelumab (19)
Number of patients	37	137	94	30
Objective response: N (%)	9 (24.3)	15 (10.9)	13 (13.8)	3 (10.0)
Complete response: N (%)	2 (5.4)	8 (5.8)	1 (1.1)	0
mPFS (months)	4.1	2.1	2.3	2.0
mOS (months)	11.5	11.7	10.1	13.9

Even though anti–PD-1/L1 therapies in HPV^+^ cancers are effective, the benefit is limited to approximately 15% of patients in monotherapy, and only a few patients achieved a long-term remission. Therefore, the development of a novel combination in order to extend the clinical efficacy of anti–PD-1/L1 is of great interest.

Altogether, the combination of anti–PD-1/L1 therapy with an antitumor vaccine gains serious consideration in HPV^+^ cancers. In the present VolATIL clinical trial, we will evaluate the clinical interest and immunological efficacy of a treatment combining the UCPVax with anti–PD-L1 therapy (atezolizumab) in patients with HPV^+^ cancers (anal canal, cervix and vulvar, and oropharyngeal cancers) by evaluation of the objective response rate (ORR) at 4 months according to iRECIST criteria. We will also explore, as a secondary objective, the feasibility of specific tumor-infiltrating lymphocytes (TIL) production.

## 2 Methods and Analysis

### 2.1 Design

VolATIL study is a multicenter phase II trial to evaluate the combination of atezolizumab and UCPVax in advanced HPV^+^ cancer patients, refractory to at least one line of systemic chemotherapy. The study was developed by the “National Institute of Health and Medical Research (INSERM), Unit 1098” and “Clinical Investigational Center (CIC) 1431”. The study is sponsored by the CHU of Besançon and supported by INCa (French National Cancer Institute) and ROCHE. The data management is undertaken by the “Methodology and Quality of Life Unit in Oncology”* from the University Hospital of Besançon. The trial is registered on the clinicaltrials.gov database (NCT03946358).

The study has received a favorable opinion from the Ethic Committee “Sud-Est IV” and the authorization from the French National Agency for the Safety of Medicines and Health Products and will be conducted in accordance with the Declaration of Helsinki and the Good Clinical Practice.

* http://www.umqvc.org/en/index.html

### 2.2 Study Objectives

The main objective of this clinical trial is to assess the efficacy of the strategy combining UCPVax and atezolizumab in patients with advanced refractory HPV^+^ cancer by evaluation of the ORR at 4 months according to iRECIST.

The secondary objectives are the following:

- OS
- Progression-free survival (PFS)
- Health-related quality of life (QoL)
- Safety of atezolizumab in combination with UCPVax
- Ability of UCPVax and atezolizumab to promote the activity of antigen specific T lymphocytes in the peripheral blood
- Ability of UCPVax and atezolizumab to enhance the tumor cell infiltration by specific lymphocytes (tumor-infiltrating lymphocytes, TIL)
- Feasibility of TIL expansion in HPV^+^ cancer patients
- Research of biomarkers

### 2.3 Patient Selection

The study population consists of patients with HPV^+^ (defined by p16^+^ by immunohistochemistry or HPV genotyping) cancer, locally advanced or metastatic, and refractory to systemic chemotherapy, corresponding to one of the following selected types:

- Carcinoma of the anus
- Carcinoma of the head and neck
- Carcinoma of the cervix or vulva

Patients should have an ECOG-PS <2 and adequate organ function.

The main inclusion and exclusion criteria are listed in **Table 2**.

**Table 2:** Main inclusion and exclusion criteria.

***Inclusion criteria*** ➢ Histologically confirmed HPV^+^ cancers, defined by p16^+^ (IHC) or HPV genotyping (HPV genotyping mandatory for non-squamous and adenocarcinoma, because p16 is not validated as a surrogate marker), corresponding to one of the following selected types: –Carcinoma of the anus –Carcinoma of head and neck –Carcinoma of cervix or vulva ➢ Locally advanced or metastatic disease ➢ Patient treated by a first line of anti-cancer standard treatment performed in advanced or metastatic disease ➢ Age ≥18 and ≤75 years old ➢ ECOG-PS <2 ➢ Signed written informed consent ***Exclusion Criteria*** Non-eligible to a clinical trial: ➢Patients previously exposed to anti-tumor immunotherapy as anti–PD-1, anti–PD-L1, or anti-CTLA4 agent or any immune therapy (HPV vaccination is allowed) ➢Diagnosis of additional malignancy within 3 years prior to the inclusion with the exception of curatively treated basal cell carcinoma of the skin and/or curatively resected *in situ* cervical or breast cancer ➢Patient with any medical or psychiatric condition or disease, which would make the patient inappropriate for entry into this study ➢Current participation in a study of an investigational agent or in the period of exclusion ➢Patient under guardianship, curatorship, or under the protection of justice Cancer-specific exclusion criteria: ➢Uncontrolled pleural effusion, pericardial effusion, ascites, or symptomatic fistula ➢Uncontrolled tumor-related pain: exposing patients to risk of exposure to corticoids or iterative hospitalizations. Symptomatic lesions amenable to palliative radiotherapy should be treated prior to inclusion. Patients should be recovered from the effects of radiation. There is no required minimum recovery period ➢Known active central nervous system metastases and/or carcinomatous meningitis. Subject with previously treated brain metastases and with radiological and clinical stability are allowed Non-eligible to treatment: ➢Inadequate organ functions: known cardiac failure of unstable coronaropathy, respiratory failure, or uncontrolled infection or another life-risk condition ➢Active or chronic hepatitis B or C and/or HIV-positive (HIV 1/2 antibodies patients), or a known history of active Tuberculosis bacillus ➢Any immunosuppressive therapy (i.e., corticosteroids >10 mg of hydrocortisone or equivalent dose) within 14 days before the planned start of study therapy ➢Active autoimmune disease that has required a systemic treatment in the past 2 years (i.e., corticosteroids or immunosuppressive drugs). Replacement therapy (e.g., thyroxine and insulin) is allowed ➢Active or history of autoimmune disease or immune deficiency, including, but not limited to, myasthenia gravis, myositis, autoimmune hepatitis, systemic lupus erythematosus, rheumatoid arthritis, inflammatory bowel disease, antiphospholipid antibody syndrome, Wegener granulomatosis, Sjögren syndrome, Guillain–Barré syndrome, or multiple sclerosis (see Annex 5 for a more comprehensive list of autoimmune diseases and immune deficiencies) with the following exceptions: ➢Patients with a history of autoimmune-related hypothyroidism who are on thyroid replacement hormone are eligible for the study ➢Patients with controlled type 1 diabetes mellitus who are on an insulin regimen are eligible for the study ➢Patients with eczema, psoriasis, lichen simplex chronicus, or vitiligo with dermatologic manifestations only (e.g., patients with psoriatic arthritis are excluded) are eligible for the study provided that all of following conditions are met: - Rash must cover ≥10% of body surface area - Disease is well controlled at baseline and requires only low-potency topical corticosteroids - No occurrence of acute exacerbations of the underlying condition requiring psoralen plus ultraviolet A radiation, methotrexate, retinoids, biologic agents, oral calcineurin inhibitors, or high potency or oral corticosteroids within the previous 12 months ➢Prior allogeneic bone marrow transplantation or prior solid organ transplantation ➢History of severe allergic, anaphylactic, or other hypersensitivity reactions to chimeric or humanized antibodies or fusion proteins ➢Known hypersensitivity or allergy to Chinese hamster ovary cell products or any component of atezolizumab formulation ➢History of idiopathic or secondary pulmonary fibrosis (history of radiation pneumonitis in the radiation field fibrosis is permitted), or evidence of active pneumonitis requiring a systemic treatment with 28 days before the planned start of study therapy ➢Major surgical procedure other than for diagnosis within 4 weeks prior to initiation of study treatment, or anticipation of need for a major surgical procedure during the course of the study ➢Severe infection within 4 weeks prior to initiation of study treatment, including, but not limited to, hospitalization for complications of infection, bacteremia, or severe pneumonia ➢Treatment with therapeutic oral or IV antibiotics within 2 weeks prior to the initiation of study treatment. Patients receiving prophylactic antibiotics (e.g., to prevent a urinary tract infection or chronic obstructive pulmonary disease exacerbation) are eligible for the study ➢Patients under chronic treatment with systemic corticoids or other immunosuppressive drugs (prednisone or prednisolone ≤10 mg/day is allowed) for a period of at least 4 weeks and whose treatment was not stopped 1 week prior to the start of the study treatment ➢Patient with intra-alveolar hemorrhage, pulmonary fibrosis or uncontrolled asthma, or chronic obstructive disease (COPD), defined as at least one hospitalization within 4 months prior to enrollment or as at least three exacerbations during the last year prior to enrollment ➢Patients requiring oxygen therapy ➢Patients with LEVF <40% ➢Hospitalization for cardiovascular or pulmonary disease within 4 weeks prior to enrollment ➢Receipt of a live, attenuated vaccine within 4 weeks prior to inclusion or anticipation that such a live, attenuated vaccine will be required during the study –Note: Patients must agree not to receive live, attenuated influenza vaccine (e.g., FluMist^®^) within 28 days prior to randomization, during treatment, or within 5 months following the last dose of atezolizumab ➢Inadequate hematology function: Lymphocyte count at baseline <800/mm^3^; neutrophil count <1,500/mm^3^; platelets <100,000/mm^3^; hemoglobin <9 g/dl ➢Inadequate hepatic function: bilirubin 2.5-fold ULN, AST/ALT 2.5-fold ULN, or 5-fold ULN with liver metastasis, international-normalized thromboplastin time ratio >1.5 ➢Inadequate renal function: MDRD CrCl <40 ml/min ➢Other inadequate laboratory values: serum albumin <30 g/L; troponin > ULN; BNP > ULN. ➢Active alcohol or drug abuse.

### 2.4 Treatments

#### 2.4.1 Atezolizumab

Patients will receive atezolizumab every 3 weeks, at fixed dose of 1,200 mg in 60-min intravenous (IV) infusion before UCPVax injection, until disease progression or unacceptable toxicity or maximum of 2 years. If atezolizumab is well tolerated at the first cycle, atezolizumab perfusion can be reduced to 30 min in the following cycles. No dose adjustment is required. No premedication treatment is required before infusion of atezolizumab.

#### 2.4.2 UCPVax

UCPVax is a telomerase-derived cancer vaccine developed by our academic research network at Besançon (Pr Adotevi, UMR1098 INSERM/EFS/UFC) and patented (n° EP: 12305319 and n° US: 61/621.075) by a consortium including the Regional University Hospital of Besançon and the University of Bourgogne Franche-Comté.

The Good Manufacturing Practice (GMP) drug substance of UCPVax was produced by PolyPeptide biotech (Strasbourg, France). Clinical grade peptide production and formulation will be performed according to GMP preparation and compliance by the Pharmacy Department of the University Hospital of Besançon like in the previous trial in lung cancer (Clinical Trial NCT02818426).

**Figure 1:**
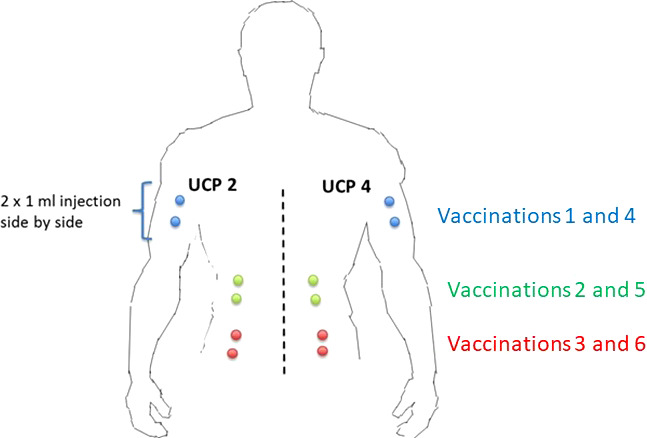
Injection sites of each peptide. The two UCPVax peptides, UCP2 and UCP4 at 1 mg/ml (combined with Montanide ISA51 as adjuvant), will be injected subcutaneously in two separate sites (one site per peptide) at days 1, 8, 15, 29, 36, and 43 (priming phase) following by boost vaccinations: every 6 weeks for the two first boosts (W13 and W19) and every 9 weeks afterward (W28, W37, and W46).

The two UCPVax peptides, UCP2 and UCP4 at 1 mg/ml (combined with Montanide ISA51 as adjuvant), will be injected subcutaneously in two separate sites (one site per peptide) (**Figure 1**) at days 1, 8, 15, 29, 36, and 43 (priming phase) following by boost vaccinations: every 6 weeks for the two first boosts (W13 and W19) and every 9 weeks afterward (W28, W37, and W46).

### 2.5 Study Description

#### 2.5.1 Therapeutic Sequence

**Figure 2:**
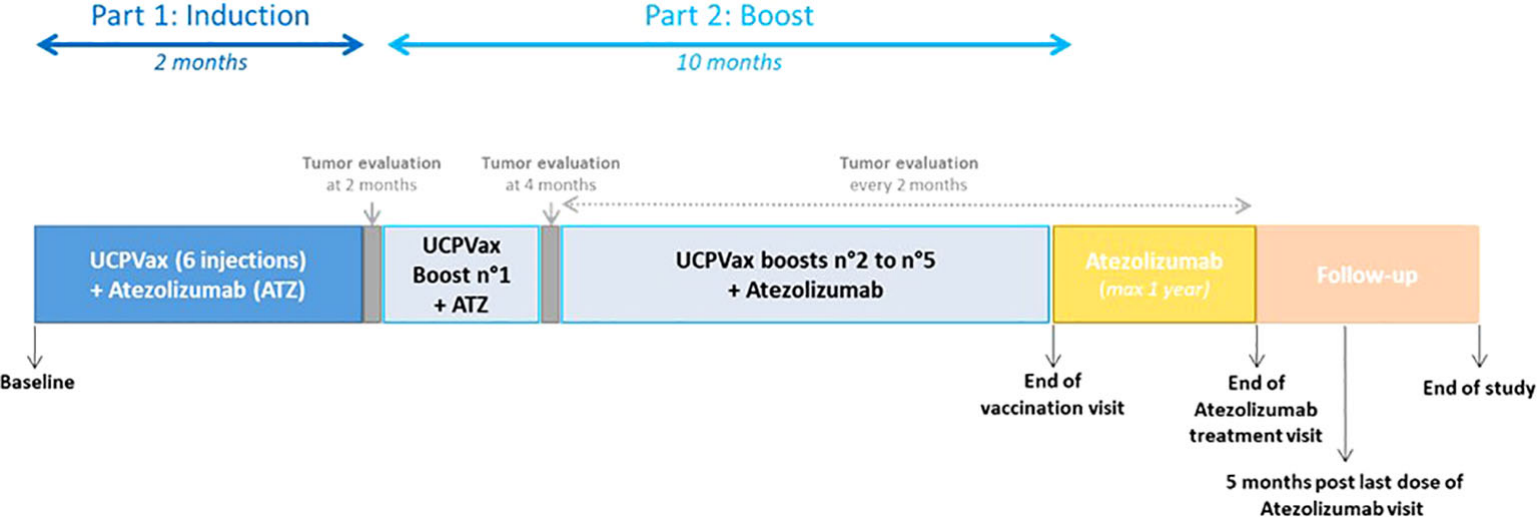
Therapeutic sequences. **Part 1** Induction phase: Atezolizumab (1,200 mg IV) will be administrated every 3 weeks starting at day 1. UCPVax will be injected subcutaneously at weeks 1, 2, 3, 5, 6, and 7. The two peptides included (UCP2 and UCP4) in UCPVax will be injected subcutaneously in separate sites (one site per peptide) after emulsion in the adjuvant (Montanide ISA-51 VG). **Part 2**: Boost phase of UCPVax+. Atezolizumab: Atezolizumab will be administered at the standard dose of 1,200 mg IV every 3 weeks. UCPVax boosts vaccine will be performed every 6 weeks for the two first boosts (W13 and W19) and then every 9 weeks thereafter (W28, W37, and W46). **Part 3**: Atezolizumab in monotherapy will be administrated every 3 weeks until disease progression or unacceptable toxicities for a maximum of 1 year since the last vaccine.

Initial assessments will be performed at baseline, within 28 days prior to the first administration of the combination atezolizumab and UCPVax. Treatment will include three phases (**Figure 2**):

##### - Part 1: Induction phase of UCPVax + Atezolizumab

Atezolizumab, 1,200 mg IV, will be administrated every 3 weeks starting at day 1. UCPVax will be injected subcutaneously (according to previous phase I/II study) at weeks 1, 2, 3, 5, 6, and 7. The two peptides included (UCP2 and UCP4) in UCPVax will be injected subcutaneously in separate sites (one site per peptide) after emulsion in the adjuvant (Montanide ISA-51 VG). Tumor assessment will be made according to iRECIST criteria every 8 weeks with a total body CT scan (if progressive disease, it could be confirmed by another total body CT scan 4 to 8 weeks after the precedent).

##### - Part 2: Boost phase of UCPVax + Atezolizumab

Only patients with stable, partial, or complete response at disease evaluation visit at 4 months will be included to Part II. Atezolizumab will be administered at the standard dose of 1,200 mg IV every 3 weeks. UCPVax boosts vaccine will be performed every 6 weeks for the two first boosts (W13 and W19) and then every 9 weeks thereafter (W28, W37, and W46).

##### - Part 3: Atezolizumab Monotherapy

Atezolizumab in monotherapy will be administrated every 3 weeks until disease progression or unacceptable toxicities for a maximum of 1 year since the last vaccine.

#### 2.5.2 Evaluation, Laboratory Tests and Follow-Up

For the primary end-point analysis, a total body CT scan will be performed at baseline, and every 8 weeks until progression. Additional total body CT scan could be performed if clinically indicated.

For immunological safety assessment, three blood samples will be collected:

- Sample N°1: At inclusion visit before the first treatment
- Sample N°2: At week 5 (before vaccination n°4)
- Sample N°3: At 2 months from inclusion

In case of immune-related adverse event, these analyses will be performed:

- Immunological blood analysis: IgG, IgE, circulating antibodies (ANA and TPO), CRP, and ferritin. Serum (one dry tube, 6 ml) will be frozen for the measure of anti-telomerase peptide (anti-UCP) antibodies.
- Cytokines release measurement (one EDTA tube, 6 ml): Plasma will be frozen for analysis of IL-1b, IL-6, IL-8, IL-2, TNFα, IFN-g, IL-17, and sCD25.

For immunomonitoring analysis, four blood samples will be collected at the following:

- Sample N°1: at baseline
- Sample N°2: at 2 months from inclusion
- Sample N°3: at 4 months from inclusion
- Sample N°4: at the end of vaccine visit

Biomonitoring Blood Samples: Six 6-ml EDTA tubes for PBMC collection, one 6-ml EDTA tubes for plasma collection, and one 6-ml EDTA tube for ctDNA collection will be collected for analysis.

#### 2.5.3 Biological Analyses

Specific anti-tumor immune responses and HPV circulating DNA analysis will be performed in collaboration with UMR1098 INSERM/EFS/UFC team and with the Cellular and Molecular Laboratory and the Pathological Laboratory of the University Hospital of Besançon

- Immunohistochemistry analysis of anti-tumor responses: Immunohistochemistry analysis will be performed to evaluate p53, p16, and anti-tumor responses to: Class I CMH, Foxp3, pSTAT3, T-bet and immune infiltrates, CD8, and GRZM. This analysis will be performed on tumor samples.
- Genotyping: Genotyping will be performed on tumor samples obtained by biopsies at diagnosis. This analysis will be performed to characterize the genomic profile of cancers and to determine the influence of the neoantigen charge on the combination therapy. If the tumor sample is sufficient, RNAseq analysis will be performed to determine biomarkers that predict the efficacy of the combination therapy. HPV genotyping will be carried out using the INNO-LiPA technique. In addition, a search for the viral genome integration *via* E6, E7, and E2 proteins will be carried out.
- Quantification of circulating HPV DNA in plasma by qPCR targeting E6 and E7 will be done before and during treatment.
- Peripheral blood immune phenotyping and plasma-derived biomarkers
  - Monocytes and MDSC will be phenotyped by flow cytometry
  - Regulatory T lymphocytes (CD3/CD4/CD25/Foxp3/CD45RA, CD15s, and CD127)
  - Immune checkpoint and costimulatory molecules will be analyzed on CD8 and CD4 T-cell lymphocytes (PD-1, TIM3, TIGIT, LAG3, CD226, OX40, 41BB, CTLA4, and CD26)
  - Angiogenic-related growth factors will be monitored in the plasma of included patients at baseline and 2 months after treatment initiation
- Assessment of antigen-specific T cells
  - Peripheral blood mononuclear cells will be exposed to E6, E7, and hTert-derived peptides. CD4- and CD8-specific T-cell IFN-γ production will be monitored by ELISpot IFN-γ. Similar experiments will be performed using TIL.

These experiments will allow us to determine the presence of antigen-specific T cells before and after treatment as well as the ability of combined vaccination and atezolizumab therapy to enhance the diversity of immune responses (epitope spreading).

#### 2.5.4 Tumor Samples Collection

- A fresh tumor biopsy for TIL production proof of concept will be performed and centralized at baseline and at 2 months. Collection of tumor biopsies will be guided by ultrasound or CT scan. Methods for TIL culturing were the same as those used by the Rosenberg group (25).
- Tumor samples obtained by biopsies or surgery at diagnosis will be centralized for the translational research program.

#### 2.5.5 Quality-of-Life Assessment

A health-related QoL EORTC QLQ-C30 questionnaire will be collected:

- at inclusion,
- every 8 weeks until the end of treatment visit, and
- at the end of vaccine visit.

#### 2.5.6 Data Analysis

For each patient enrolled in VolATIL study, the investigators will document all required data in the corresponding source documents. These data will then be entered in the electronic case report form (eCRF), which will be accessible only to authorized persons ***via*** a secured web connection. One eCRF will be created for each patient. The investigator has the responsibility for its completion, proof reading, and its approval after the final verification for the authenticity and accuracy of all entered data. University Hospital of Besançon (Sponsor) will ensure that the study is conducted in accordance with the Good Clinical Practice guidelines and all applicable local laws and that the rights, security, and wellbeing of the patient are respected. The Sponsor will perform source document verification and validation as well as request clarification to ensure the accuracy, completeness, and reliability of data. At the end of the data handling process, a data review meeting will be held to prepare the database lock. After the database lock, data will be transferred into SAS format. Methodology and Quality of Life Unit in Oncology from the University Hospital of Besançon will produce statistical analyses.

#### 2.5.7 Statistical Considerations

VolATIL trial is a one-arm two-stage phase II design. The aim is to demonstrate that the ORR at 4 months according to iRECIST is clearly above a low rate (such as 20%) that would be not satisfactory. The following hypotheses will be considered:

- H0 (null): An ORR rate at 4 months of 20% is uninteresting
- H1 (alternative): An ORR rate at 4 months of 45% is expected

According to Simon two-stage design (optimum method) with a one-sided 2.5% type I error and power of 90%, 42 evaluable patients at 4 months will need to be included in order to test the previous hypotheses.

The hypotheses regarding an uninteresting rate of 20% are based on previous studies (17–20, 22–24, 26). An anticipated ORR rate at 4 months of 45% would be considered clinically meaningful.

**Stage 1**: After recruitment of the first 14 evaluable patients with a 4-month follow-up from inclusion,

- if three or less than three patients present an objective response at 4 months (21.4%), the treatment could be declared uninteresting. No more additional patients will be included in this arm
- if 4–14 patients present an objective response at 4 months, 28 additional patients will be enrolled in the study.

The probability to conclude for futility at the end of stage 1 whereas p = 45.0% is β1 = 6.3%. This early look interim analysis is planned to look for an early efficacy and safety data.

**Stage 2**: After recruitment of 42 evaluable patients with a follow-up of 4 months from inclusion,

- if 13 or less than 13 patients present an objective response at 4 months (≤ 31.0%), the treatment will be declared uninteresting
- if 14 or more than 14 patients present an objective response at 4 months (≥ 33.0%), the treatment will be regarded as interesting for further evaluation in a phase III trial.

Of note, the rational for H1 hypothesis is sustained by the results of HPV vaccination combined with nivolumab where an ORR of 33% was observed (27).

The probability to conclude for futility at the end of stage 2 whereas p = 45.0% is β2 = 9.3% (i.e. power = 90.7%). The probability to conclude for efficacy at the end of stage 2 whereas p = 20.0% is α2 = 2.5%. With an expected 10% rate of patients not evaluable or lost to follow-up at 4 months, it will be necessary to include (42*100)/90 = 47 patients in the study.

An Independent Data and Safety Monitoring Committee (IDMC) will be constituted and will meet for the validation of the passage from stage 1 to stage 2 for the monitoring of toxicities and also for the reviewing of SAEs every 6 months.

In parallel, given that the safety profile of UCPVax and atezolizumab combination has not been evaluated in patients with HPV-positive cancer so far, a semi-continuous monitoring for toxicity using Pocock-type boundary will be performed [probability of early stopping: 0.05, targeted dose-limiting toxicity (DLT) rate: 0.20]. Patient recruitment will be stopped and discussed with the DSMB (Data Safety Monitoring Board) at the end of stage 1 if 7 or more patients of 14 enrolled patients with a 4-month follow-up experience relevant DLT.

### 2.6 Modality of Analysis

The primary analysis will be on modified intention-to-treat (mITT) population, i.e., including all evaluable patients regardless of their eligibility and treatment received. Confirmative analyses will be conducted firstly in the ITT population (not assessable patients and patients with dropout between inclusion and 4 months will be considered as progressive) and, secondly, in the Per Protocol population defined as patients who have received at least one dose of treatment and presenting no major deviations from the protocol.

Safety analyses will be conducted in all patients who have received at least one dose of allocated treatment.

### 2.7 End of Trial

This study will end once the 47 recruited patients have performed the “5 months post last dose of atezolizumab” visit.

Every patient participating in this study can stop the trial at any moment and has no need to justify the reason for this decision. In that case, patients will pursue the standard medical care.

The reason for an early end of the study must be declared as one of the following criteria: patient’s decision, severe adverse event, protocol deviation, loss of follow-up, or death.

### 2.8 Monitoring and Safety

An “Independent Data and Safety Monitoring Committee” (IDMC) represented by a multidisciplinary team, including physicians from relevant medical disciplines and biostatistical background, is implemented to the following:

✓ ensure the safety and wellbeing of the patients exposed to study treatment through the ongoing review of safety data
✓ assure the highest integrity about operation and performance of the trial
✓ evaluate ongoing safety data to detect the possibility of an unfavorable early treatment risk. IDMC will meet for the validation of the passage from stage 1 to stage 2 (see the *Statistical considerations* section) for the monitoring of toxicities and also for the reviewing of SAEs every 6 months. IDMC will meet also in case of unexpected toxicities or as requested.

IDMC’s responsibilities are as follows:

✓ to review safety data on an ongoing basis
✓ to recommend that the clinical study be stopped early if there is strong evidence that the investigational medicinal products are harming patients
✓ to make recommendations regarding modification of the study if there is strong evidence that such change would substantially contribute to the wellbeing of patients.

Safety results and opinion of the committee will be sent to the ANSM (French Health Products Safety Agency) within 10 days after the analyses of tolerance (additional meetings will be performed in any event of a signal that may affect patients’ safety).

The IDMC will independently make its recommendations for continuation or termination of the trial to the appropriate Sponsor contact. The IDMC will maintain records of all its meetings and activities related to the study.

These records will remain confidential until completion of the final analysis at which time they will be forwarded to the Sponsor for appropriate filing.

Members will be aware of data management and quality control procedures and ensure that the data are timely, accurate, and complete.

## 3 Discussion

Current clinical trials have shown that less than one patient out of five achieved clinical responses to anti–PD-1 monotherapy. Combining such immune checkpoint inhibition treatment with vaccination might increase the number of patients achieving a clinical benefit. In this combination trial, more than 45% of objective responses are expected.

There is currently no consensus on standard of care for HPV^+^ squamous carcinoma previously exposed to platinum containing chemotherapies. Combining atezolizumab with T_H_1-promoting vaccine might constitute an alternative therapeutic option for these patients.

## Ethics Statement

The studies involving human participants were reviewed and approved by The French National Agency for the Safety of Medicines and Health Products CPP Sud-Est IV. The patients/participants provided their written informed consent to participate in this study.

## Author Contributions

Conception and design: LM, TM, DV, SK, and CB; Protocol writing: LM, TM, MR-P, and CB; Methods: AV, DV, and CB. Study promotion: MJ and MR-P; Manuscript writing: MR-P, SK, and CB; Critical lecture and final approval of the manuscript: all authors.

## Funding

Roche France provides atezolizumab. INCa (Institut National du Cancer) and Fondation ARC (Inca-ARC_13566) fund the study.

## Conflict of Interest

CB: Research grant from Bayer and Roche, advisory board for Bayer, Sanofi, MSD. FB: Novartis, Seagen, Daiichi Sankyo, Pierre Fabre, Astra-Zeneca, Clovis. SK: reports a consulting or advisory role for Ipsen, Incyte, Boehringer Ingelheim, Sanofi and BeiGene and has received research funding from Pfizer, Roche, Novartis, Bristol-Myers Squibb, Boehringer Ingelheim and Sanofi.

The remaining authors declare that the research was conducted in the absence of any commercial or financial relationships that could be construed as a potential conflict of interest.

## Publisher’s Note

All claims expressed in this article are solely those of the authors and do not necessarily represent those of their affiliated organizations, or those of the publisher, the editors and the reviewers. Any product that may be evaluated in this article, or claim that may be made by its manufacturer, is not guaranteed or endorsed by the publisher.

## Abbreviations

ECOG-PS, Eastern Cooperative Oncology Group-performance status; eCRF, electronic case report form; HNSCC, Head and Neck Squamous Cell Carcinoma; HPV, human papilloma virus; HR, hazard ratio; IDMC, Independent Data and Safety Monitoring Committee; NSCLC, Non–Small Cell Lung Cancer; ORR, objective response rate; OS, overall survival; PD1/PD-L1, programmed cell death protein-1 immune checkpoint and its ligand; PFS, progression-free survival; QoL, health-related quality of life; SCCA, Squamous cell carcinoma of the anus.
